# Plant Development in the Garden Pea as Revealed by Mutations in the *Crd/PsYUC1* Gene

**DOI:** 10.3390/genes14122115

**Published:** 2023-11-23

**Authors:** Ariane Gélinas-Marion, Morgane P. Eléouët, Sam D. Cook, Jacqueline K. Vander Schoor, Steven A. G. Abel, David S. Nichols, Jason A. Smith, Julie M. I. Hofer, John J. Ross

**Affiliations:** 1 School of Natural Sciences, University of Tasmania, Sandy Bay, Hobart 7001, Australia; ariane.gelinasmarion@utas.edu.au (A.G.-M.); jacqueline.vanderschoor@utas.edu.au (J.K.V.S.); steven.abel@bayer.com (S.A.G.A.); jason.smith@utas.edu.au (J.A.S.); 2 Institute of Biological, Environmental and Rural Sciences, Aberystwyth University, Plas Gogerddan, Aberystwyth SY23 3EE, UK; morgane.eleouet@yahoo.fr; 3 Department of Chemistry, Umea University, Linnaeus vag 10, Kemi A3, 901 87 Umea, Sweden; sam.cook.a@gmail.com; 4 Central Science Laboratory, University of Tasmania, Sandy Bay, Hobart 7001, Australia; d.nichols@utas.edu.au

**Keywords:** pea, *YUCCA*, *Crispoid*, auxin, compound leaves, organ initiation, plant development, auxin inactivation

## Abstract

In common with other plant species, the garden pea (*Pisum sativum*) produces the auxin indole-3-acetic acid (IAA) from tryptophan via a single intermediate, indole-3-pyruvic acid (IPyA). IPyA is converted to IAA by PsYUC1, also known as Crispoid (Crd). Here, we extend our understanding of the developmental processes affected by the *Crd* gene by examining the phenotypic effects of *crd* gene mutations on leaves, flowers, and roots. We show that in pea, Crd/PsYUC1 is important for the initiation and identity of leaflets and tendrils, stamens, and lateral roots. We also report on aspects of auxin deactivation in pea.

## 1. Introduction

Auxin biosynthesis mutants have implicated auxin in plant development [1,2,3,4,5,6,7,8,9,10], although relatively few eudicot species have been investigated in this way. The garden pea (*Pisum sativum*), for example, has not been fully exploited, despite its history as a model species for auxin research and the fact that it differs markedly from other model species in the structure of its leaves.

Pea has compound leaves, comprised of stipules, leaflets, and tendrils. The leaflets and tendrils, known collectively as pinnae, are borne oppositely on the leaf rachis (Figure S1). Pea leaf development is heteroblastic: the first two nodes of the main stem bear scale leaves, subsequent nodes produce leaves with only one pair of leaflets and one tendril, and later nodes typically bear leaves with additional pinna pairs.

In the pea lines used here, after the production of at least eleven vegetative nodes, compound inflorescences [11] are produced in the leaf axils. The flowers, borne on the secondary inflorescences, are bilaterally symmetrical [12] and consist of three petal types: banner, alae, and carinae (Figure S1), in contrast to Arabidopsis and petunia flowers, which have only one petal type. There is also a major difference in the development of pea flower primordia. In the ABCDE or ABCE model of floral organ identity, separate primordia specify each flower’s organ type (the four whorls) [13,14], whereas in pea, the petals and five of the ten stamens arise from common primordia [15,16].

As in other species [6,10,17], the main auxin biosynthesis pathway of pea appears to involve just two steps: tryptophan to indole-3-pyruvic acid (IPyA), and IPyA to indole-3-acetic acid [IAA]. These two steps are catalysed by the TRYPTOPHAN AMINOTRANSFERASE OF ARABIDOPSIS (TAA)/TRYPTOPHAN AMINOTRANSFERASE RELATED (TAR) and YUCCA (YUC) enzyme families, respectively [17]. Previously, we have shown that a mutation in the pea *PsTAR2* gene reduces the auxin content in seeds and consequently, starch synthesis and seed size, but the direct effects of this mutation are limited to the seeds [18].

Mutations in *YUCCA* genes disrupt auxin biosynthesis [1,2,3,4,5,6,7,8,9,10,17]; however, their full range of phenotypic effects is not understood. In pea, the *PsYUC1* gene (also known as *Crd*), is required for leaflet venation patterning [19]. We previously identified four mutant alleles: a mis-sense mutation (*crd-1*), a splice site mutation (*crd-2*), a deletion mutation (*crd-3*), and a frameshift mutation (*crd-4*), and studied the physiological consequences of reduced vein formation in the *crd-4* mutant [19]. Here, we describe other major phenotypic effects of *crd* mutations and show that *Crd*/*PsYUC1* is crucial for the normal development of pea leaves, flowers, and roots. The *Crd*/*PsYUC1* gene is known to be expressed in these organs [20,21].

## 2. Materials and Methods

### 2.1. Plant Lines

The *crd*-*1* mutant was originally obtained via N-nitroso-N-ethylurea (ENU) mutagenesis in the Paloma WT-1 line [22]. It is maintained in the line JI2460. The Paloma line used here was line JI3538 (termed WT-1). The *crd-3* mutation is maintained in the line FN1522/1. It was obtained after fast neutron mutagenesis in the line JI2822 (WT-3) [23], followed by backcrossing to JI2822 three times before this study. All these lines were obtained from the John Innes Centre Pisum Germplasm collection. The *crd-4* mutation was discovered in Caméor TILLING line 905, initially obtained in order to find mutations in the gene *PsTAR1* [19]. It was either backcrossed to Caméor five times or bred onto a tall (*Le*) background (Torsdag; four backcrosses). Paloma, JI2822, and Caméor are dwarves (*le-1*). To study *crd-3* roots, the *crd-3* line was also bred onto a Caméor background (three backcrosses; see Figure captions).

### 2.2. Growth Conditions

The WT and accompanying *crd-1* and *crd-3* plants used were grown between June and August 2015 in a glasshouse in Aberystwyth, Wales, under natural conditions (13.30 to 16.30 h photoperiod and temperatures ranging from 8.6 to 18.2 °C). Mutant *crd-4* and its comparable WT plants (denoted WT-4) as well as *crd-3* plants and its comparable WT on a Caméor background were grown under controlled glasshouse conditions in Hobart, Tasmania, as previously described [19]. All node counts started with the first scale leaf as node 1.

### 2.3. Synthesis of oxIAA-Asp

The method used to synthesise oxIAA-Asp was similar to that of Kai et al. 2007 [24], and the identity of the product was confirmed using 1H and 13C NMR.

### 2.4. Extraction and LC-MS Analysis of IAA, IAA-Asp, and oxIAA-Asp

IAA and IAA-Asp were extracted and quantified from the 2 mm tips of lateral roots from *crd-3* and co-segregating WT-3 plants (Caméor background). A biological replicate (n = 5) contained 10 tips pooled (averaging 15 mg ± 1 mg) from a single plant.

Root tip tissues were extracted and derivatised as described previously, with minor adjustments [25]. Tissue was weighed and placed intact in 1 mL of sodium phosphate buffer (50 mM, pH7). To quantify the endogenous compounds, stable isotope-labelled internal standards were added; these were [^13^C_6_]-IAA (Cambridge Isotope Laboratories, Tewksbury, MA, USA) and [^15^N, ^2^H_5_]-IAA-Asp (OlChemim, Olomouc, Czech Republic). The samples were pulverised with a physcotron homogeniser (Microtech, Chiba, Japan). Aliquots (500 μL) were added to 3 mL of a cysteamine solution (0.25M) and incubated at room temperature for one hour with agitation. The pH of the samples was reduced to ~2.7 with 10 N HCl before being loaded onto a preconditioned SepPak cartridge for solid phase extraction under vacuum (with 3 mL methanol, 3 mL purified water, and 500 μL sodium phosphate buffer at pH 2.7). The samples were then washed with 2 mL of 1% acetic acid and eluted into round-bottomed flasks with 2 ml of 80% MeOH. Eluates were taken to dryness via rotary evaporation, re-suspended in 1% acetic acid (100 μL), transferred to a new Eppendorf tube, and spun at 13,000 rpm for 5 min to remove any remaining debris. Supernatants (75 μL) were transferred to auto-sampling vials and analysed promptly.

For the detection of oxIAA-Asp, young Caméor seeds (11 days past anthesis; 0.2677 g) were pulverised in 6 mL 65% isopropanol and kept at 4 °C overnight. An aliquot of 3 mL was dried and taken up in 120 μL of 1% acetic acid, transferred to an Eppendorf tube, spun at 13,000 rpm for 5 min, and the supernatant was transferred to an auto-sampling vial for analysis via UPLC-MS.

Analyses were performed using a Waters Acquity H-class UPLC system coupled to a Waters Xevo TQ triple quadrupole mass spectrometer (Waters Corporation, Milford, MA, USA). Chromatography was performed using an Acquity BEH C18 VanGuard pre-column (5.0 × 2.1 mm, 1.7 μm) and an Aquity BEH C18 analytical column (100 × 2.1 mm × 1.7 μm) (Waters Corporation). The UPLC was operated with a mobile phase consisting of 1.0% (*v*/*v*) acetic acid (Solvent A) and acetonitrile (Solvent B). The gradient ran from 5% B to 50% B over 9 min. The system was returned to its initial conditions at 9.1 min and re-equilibrated for 3 min. The flow rate was 0.35 mL/min and the column was held at 45 °C. The injection volume was 10 μL.

IAA and IAA-Asp were monitored as described previously [19]. Oxidised auxin conjugates were detected using multiple reaction monitoring (MRM) in both positive and negative electrospray ionisation modes. Electrospray ionisation was performed with a capillary voltage of 2.5 kV, with individual cone voltages and collision energies for each MRM transition. The desolvation temperature was 450 °C, the nebulising gas was nitrogen at 950 L/h, and the cone gas was nitrogen at 100 L/h. The MRM transition dwell times were 42 msec. The compound oxIAA-Asp was analysed in the negative ion mode and the main MRM transitions were precursor [M-H]^−^ (*m/z*) 305.2 to products (*m/z*) 146.1 and 189.1, with a cone voltage of 28 V and collision energies of 26 V and 16 V, respectively. This compound was also analysed in the product scan mode, over the range (*m/z*) of 50 to 350.

## 3. Results

The *Crd/PsYUC1* gene corresponds to locus Psat6g030600 on *P. sativum* Caméor genome version 1a [26]. *Crd/PsYUC1* appears to be orthologous to the *Medicago truncatula* gene, *LLS1/MtYUC1* (locus Medtr1g011630 on *M. truncatula*, A17 version 4.0), on the basis of sequence similarity and microsynteny; genes upstream and downstream of *Crd* are conserved in *Medicago*. In contrast to *Medicago*, pea leaves develop tendrils at their distal ends, so here we focus on the phenotypic effects of the *crd-1*, *crd-3*, and *crd-4* mutations [19] on pea leaves. We also examine the phenotypes of mutant flowers and roots, as these have not yet been described in legumes.

### 3.1. Effects of crd Mutations on Leaf Development

To investigate the effects of *crd* mutations on leaf development, we compared *crd-1* plants with WT-1, and concurrently, *crd-3* plants with WT-3. The principle applied was that if the same phenotypic effect was observed in both mutants, we can be confident that the *crd* mutations, not a mutation in a gene linked to *Crd*, caused that effect.

#### 3.1.1. Leaf Organ Initiation Is Reduced in *crd* Mutants

**Figure 1:**
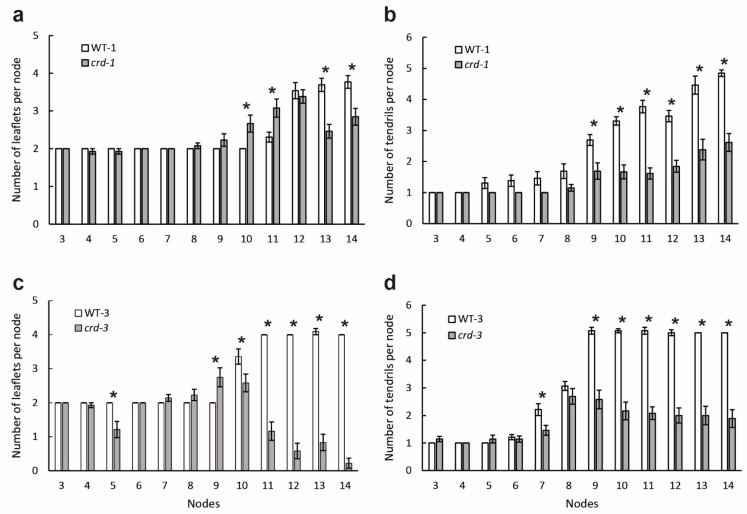
Leaf development in WT and *crd* mutant plants. (**a**) Number of leaflets in WT-1 and *crd-1* plants, for nodes 3 to 14; (**b**) number of tendrils in WT-1 and *crd-1* plants, for nodes 3 to 14. Data are shown as means ± SE; n = 13 or 14; (**c**) number of leaflets in WT-3 and *crd-3* plants, for nodes 3 to 14; (**d**) number of tendrils in WT-3 and *crd-3* plants, for nodes 3 to 14. Data are shown as means ± SE; n = 8, except for WT-3 node 14, where n = 5. Under-developed leaflets and tendrils are included. Asterisks denote significant differences at the *p* < 0.05 level.

At lower nodes on the stem (e.g., nodes 3 and 4), the leaf morphology of mutant plants was the same as WT plants (Figure 1a–d). These relatively simple leaves consisted of two leaflets and one tendril, as well as the petiole and two stipules. Nodes 1 and 2, bearing rudimentary scale leaves, were excluded from the analysis.

At progressively higher nodes, both the WT and *crd* leaves became more complex (Figure S1), but the *crd* leaves to a lesser extent. The reduced complexity in mutant plants was mainly due to a deficiency of tendrils (Figure 1b,d), but there was a deficiency of leaflets at higher nodes as well (Figure 1a,c and Figure S3).

In the comparison detailed in Table 1, *crd-3* plants formed 32% fewer identifiable leaflets per plant and 41% fewer identifiable tendrils per plant than WT-3. In mutant plants, small protuberances on the rachis (on average, five per mutant plant) were sometimes present where a leaflet or tendril was expected to develop. The total number of structures per plant (including protuberances) was 30% less in the *crd-3* mutant than in the WT (Table 1). For the *crd-1* mutant, the reductions (compared with the WT-1 plants, over nodes 13 to 19) were as follows: leaflets, 27%; tendrils, 37%; and the total number of structures per plant, 33% (Table S1). These reductions represent the extent to which mutant plants failed to develop mature pinnae and show that *Crd*/*PsYUC1* is required for each plant to initiate a full complement of leaflets and tendrils.

All mutant plants exhibited abnormalities in leaf development at most nodes, but not necessarily at all nodes.

#### 3.1.2. Leaf Organ Identity Is Altered in *crd* Mutants

A characteristic of heteroblasty in pea is that the number of tendril pairs per leaf increases before the number of leaflet pairs per leaf increases: for WT-1 and WT-3 plants, the increase in tendril number on ascension of the stem preceded that in leaflet number, by up to six nodes (Figure 1). This pattern was disrupted in the *crd-1* mutant, where increases in the number of both leaflets and tendrils began at the same node (node 8, Figure 1a,b) and in the *crd-3* mutant, although slightly less clear (node 7, Figure 1c,d). Notably, at the nodes just preceding the increase in leaflet number in the WT, mutant plants produced more leaflets than WT plants (e.g., nodes 9–11 for *crd-1*, nodes 7–9 for *crd-3;* Figure 1), while possessing fewer tendrils. The increase in leaflet number in *crd-1* plants at nodes 9–11 (Figure 1) was due to the formation of some leaflets at the second pinna position; this did not occur until higher nodes in WT plants, suggesting that organ identity at the second pinna position was affected in *crd* mutants.

Consistent with those observations, on ascension of the stem, the reduction in tendril number in the mutants, compared with their corresponding WT plants, began at a lower node than the reduction in leaflet number (Figure 1).

#### 3.1.3. Additional Effects of the *crd* Mutations on Leaf Development

**Figure 2:**
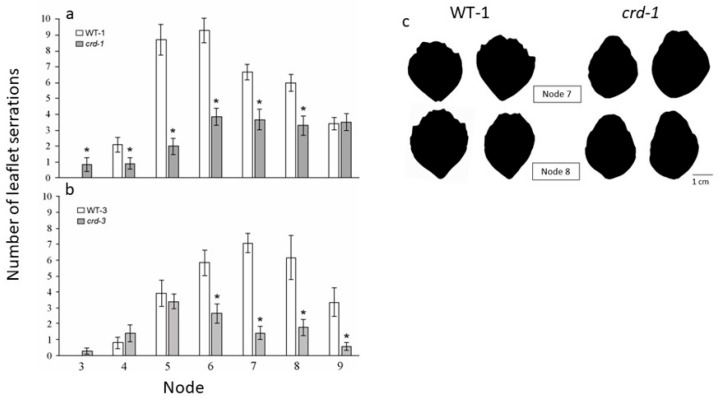
Number of serrations per leaflet at nodes 3 to 9 of (**a**) WT-1 and *crd-1* plants and (**b**) WT-3 and *crd-3* plants. Data are means ± SE. Asterisks denote a significant difference between the WT and comparable mutant (*p* < 0.05). n > 9. (**c**) Photocopies of WT and *crd-1* leaflets from nodes 7 and 8.

An obvious characteristic of mutant leaves (at approximately node 6 and above) was the reduced degree of leaflet marginal serration. For example, the leaflet serration number was significantly lower in *crd*-*1* than in WT-1 at nodes 4 to 8, and lower in *crd-3* than in WT-3 from nodes 6 to 9 (Figure 2). In addition, the tendrils of mutant plants exhibited a reduced degree of curvature compared with the tendrils of corresponding WT plants (Figure S3). The *crd* mutations reduced leaflet venation density in all leaves, regardless of ontogenetic stage, as documented previously [19] in photosynthetic studies conducted on node 4 leaves. The vast majority of leaflets that were initiated on mutant plants developed fully (apart from a small minority of protuberances and hair-like structures), and there was no consistent effect of the *crd* mutations on leaflet size (Figure S2a).

The *crd-1* and *crd-3* mutations reduced the rate of node formation of the main stem (Figure S2b).

### 3.2. crd Mutants Exhibit Floral Abnormalities

**Figure 3:**
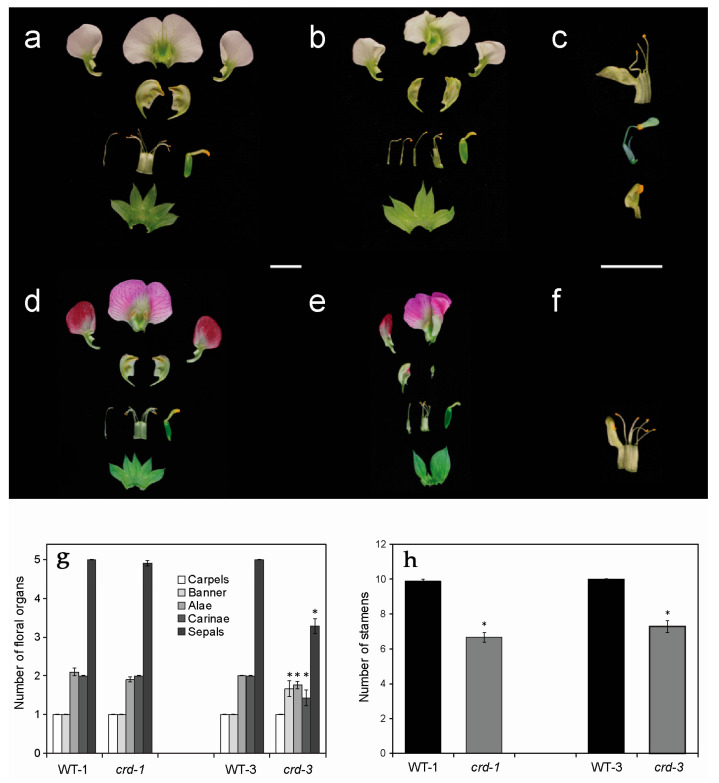
Flower structure of the *crd* mutants, compared to their respective WTs. Flowers are shown deconstructed; see Figure S1: (**a**) WT-1 flower, note the single free stamen; (**b**) *crd-1* flower with missing and unfused stamens; (**c**) *crd-1* stamens fused with petal-like structures; (**d**) WT-3 flower; (**e**) *crd-3* flower with missing petals, stamens, and sepals; (**f**) *crd-3* stamens fused with a petal-like structure; (**g**) average number of floral organs in the different genotypes; and (**h**) average number of stamens in the different genotypes. Data are means ± SE. Asterisks indicate significant differences between WT and *crd* plants (*p* < 0.05; n = 21). Scale bars = 10 mm.

As with leaf development, to investigate the effects of the *crd* mutations on flower development, we again compared the *crd-1* and *crd-3* mutations with their respective WTs. Both mutants exhibited obvious flower deformities. Petals were often asymmetrical, misshapen, and wavy, and one of the two alae (wings) was sometimes missing (Figure 3b,e). The number of stamens was consistently reduced (Figure 3h) and some stamens were not fused into a tube, unlike in the WT (Figure 3a,b). On the other hand, there was some fusion between stamens and petal-like structures (Figure 3c). The carpel number (one) was unaffected in mutant flowers (Figure 3g).

### 3.3. A Further crd Mutation, crd-4, Affects Plant Development Similarly to crd-1 and crd-3

**Figure 4:**
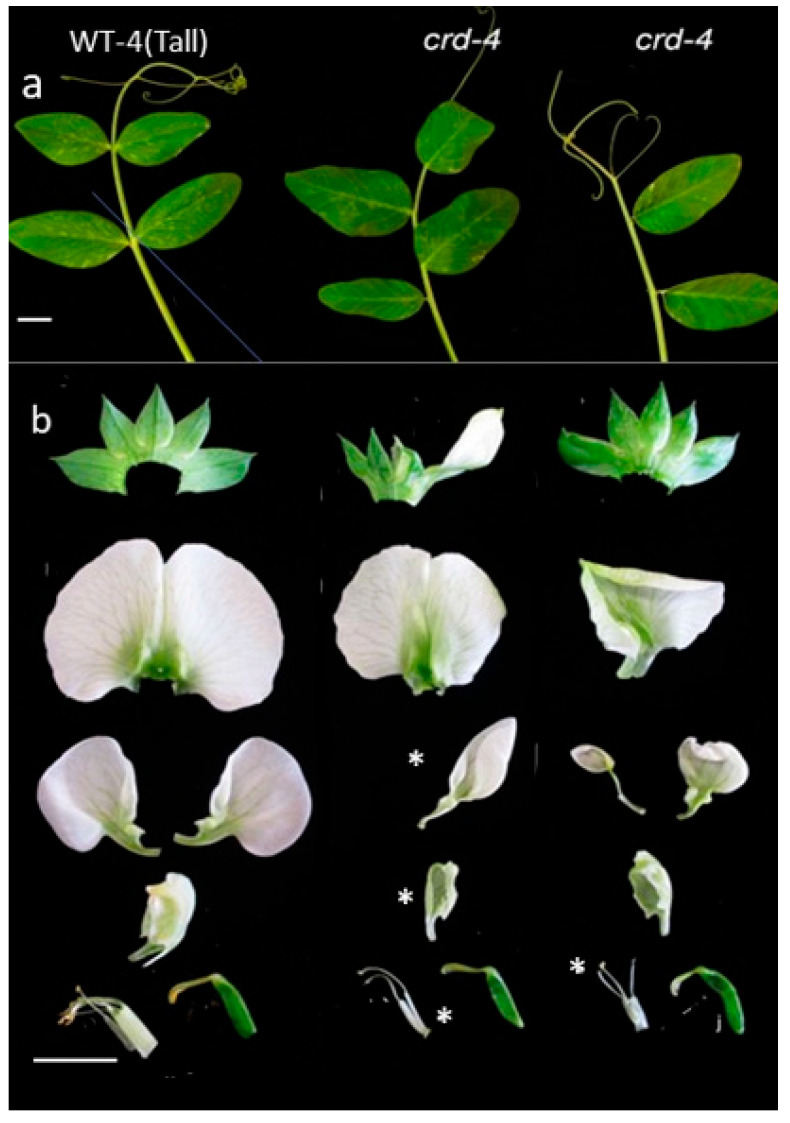
Leaves and flowers of the *crd-4* mutant and comparable WT plants. Both genotypes are in a tall (*L*e) background. (**a**) Leaves from node 12; (**b**) deconstructed flowers. From top to bottom: sepals, banner, alae, carinae, stamens, (left), and carpel. One free stamen is visible in the WT-4 case. * indicates missing organs. Scale bar = 10 mm.

In the course of this study, we identified another *crd* mutation, termed *crd-4*. This mutation was initially observed in the dwarf line, Caméor. We subsequently introgressed *crd-4* into a tall background by crossing to the cultivar Torsdag. This resulted in *crd-4* and “WT-4(tall)” plants in a *Le* background. The *Le* product catalyses the formation of bioactive gibberellin, resulting in an increased internode elongation, compared with the dwarf lines, WT-1, WT-3, and Caméor [27]. In the tall background, *crd-4* mutant plants exhibited similar phenotypes to those detailed above for *crd-1* and *crd-3*. For example, compared with WT-4 (tall), leaflets and tendrils were reduced in number, and the tendrils coiled to a lesser extent. Furthermore, *crd-4* leaflets were sometimes not borne oppositely (Figure 4; this was also observed in the *crd-1* and *crd-3* mutants described in Section 3.1). Mutant *crd-4* flowers were deformed, with some petals and stamens missing (Figure 4). When present, distal flowers on inflorescences were even more severely deformed than proximal flowers (Figure S4). This last effect is most likely attributable to *crd-4*, although we note that introgression does involve the introduction of genes linked to the gene of interest.

These observations on *crd-4* confirm that the developmental defects described above for mutants *crd-1* and *crd-3* are attributable to the *crd* mutations. They also indicate that the *crd* mutant phenotypes are general in occurrence and not restricted to a particular environment, since the *crd-4* and WT-4 plants were grown under different conditions from those used for the *crd1*/*crd3* experiments. Furthermore, mutant *crd* phenotypes are not restricted to a dwarf genetic background, being observed also in tall plants (Figure 4).

### 3.4. Effects of crd Mutations on Root Development

**Figure 5:**
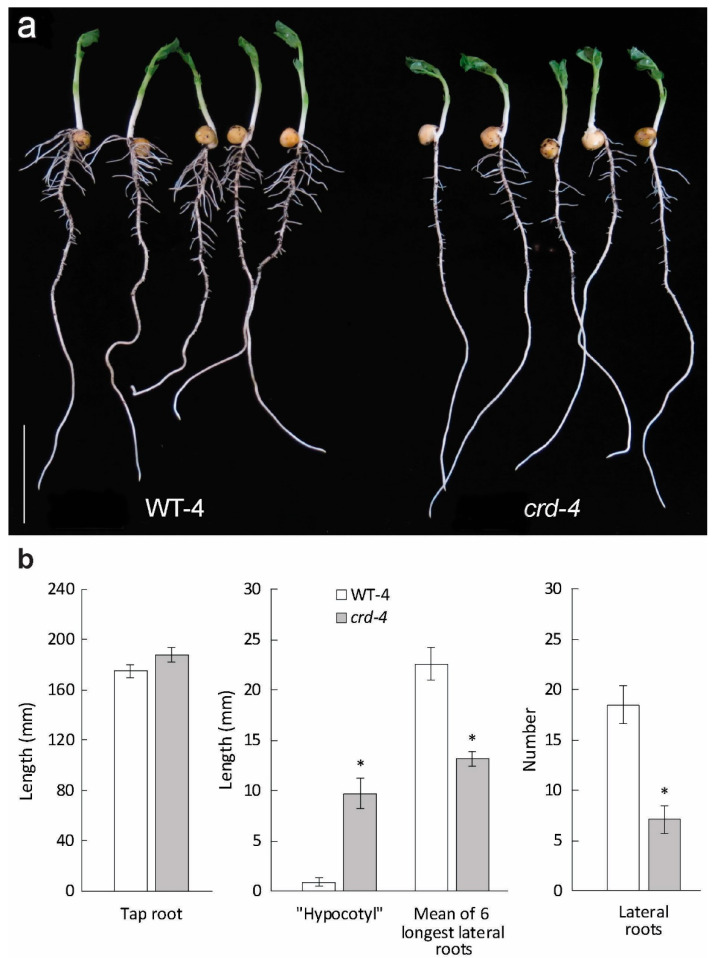
Effects of *crd-4* on root development. (**a**) 11 day-old *crd-4* and WT-4 (Caméor background) seedlings. Scale bar = 50 mm; (**b**) root phenotypes of 11 day-old *crd-4* and WT-4 (Caméor background) seedlings. “Hypocotyl” refers to the length between the cotyledons and the uppermost lateral root. Results are means ± S.E. (n = 10) and asterisks denote significant differences at the *p* < 0.01 level.

For analysis of root development, the *crd-4* mutant in the Caméor background was used, and for additional comparisons, the *crd-3* allele was introgressed into the Caméor background. In 11-day-old *crd-4* seedlings, lateral roots were less numerous and shorter when compared with WT-4 (Caméor) plants, while taproot length was not significantly different between *crd-4* and WT-4 (Caméor) (Figure 5a,b). Similar observations were made using the *crd-3* mutant and WT-3 (Caméor) at a later developmental stage (leaf 5 fully expanded; Figure S5). The “hypocotyl”, defined here as the taproot between the cotyledons and the uppermost lateral root, was longer in both mutants than in their corresponding WTs (Figure 5a,b). Again, that two independent *crd* mutations both affecting the lateral root outgrowth indicates a direct effect of Crd/PsYUC1 on root development.

We reported previously (19) that in shoots, the *crd-4* mutation reduces the content of the IAA conjugate, IAA-aspartate (IAA-Asp), to a greater extent than that of IAA itself. Here, we report a similar phenomenon for the *crd-3* mutation in lateral root tips (Figure S6). We also identified the deactivation product of IAA-Asp, oxIAA-Asp (in immature pea seeds; Figure S7) by comparing chromatograms from the extract with those from an authentic standard synthesised in our laboratory (Figures S7 and S8). This confirms a recent report for germinating seedlings [28]. We also report that the Caméor gene, Psat2g146920, likely encodes the 2-oxidation enzyme (designated PsDAO1), which is responsible for the two-oxidation step that produces oxIAA-Asp (Figures S9 and S10) [29,30].

### 3.5. Pisum YUC Genes

A BLASTp search of the pea Cameor v1a genome using PsYUC1 (Genbank Accession number ADP88696.2) as a query identified 25 flavin-binding monooxygenases with a bitscore match greater than 200. Of these, nine correspond to the YUC-encoding genes that have been documented previously. Tivendale et al. [21] reported on two of these, *PsYUC1/Crd* (Psat6g030600) and *PsYUC2* (Psat5g023680), six (Psat1g042120, Psat5g082680, Psat3g053880, Psat3g024720, Psat5g060240, and Psat3g024760) were noted by Davis et al. [31], and one more (Psat6g034040) by Kaur et al. [32]. The gene *PsYUC2* of Tivendale et al. [21] (Genbank Accession number HQ439908.1) is the same as the *PsYUC6-2* of Kaur et al. [30] and differs from the *PsYUC2* of Kaur et al. [32]. The *PsYUC1*/*Crd* gene appears to be the same as *PsYUC4* of Chabikwa et al. [33]. Variations in *PsYUC* nomenclature are shown in Table S2.

## 4. Discussion

The fact that pea produces compound leaves allowed us to examine the role of the *Crd* gene and auxin biosynthesis on organ initiation within those leaves. Using different *crd* mutant alleles and their corresponding WT lines, we found that *crd* mutants produced fewer leaflets and tendrils than the WTs, indicating that *Crd*/*PsYUC1* promotes the initiation of lateral organs on the developing leaf axis.

We also examined the effect of *Crd*/*PsYUC1* on the eventual fate of pinna primordia as either a leaflet or a tendril. Our results indicate an important role for this gene in that process because at nodes just before the change to two pinna pairs in WT plants, *crd* mutant plants produced more leaflets, but fewer tendrils, than the WT. This was the case even though the overall tendency throughout the life of the plant was for the mutants to form fewer leaflets than WT plants. This evidence supports models in which auxin favours tendril formation over leaflet formation and auxin deficiency favours leaflet formation over tendrils, e.g., [34].

Recently, the effects of *YUC* mutations on compound leaves have also been reported for *Medicago truncatula* [1] and *Fragaria vesca* [5]. These species are not closely related to each other, but both produce trifoliate leaves. Despite the name of the mutant, *lateral leaflet suppressor 1*, it was reported that in *Medicago*, the *lls1-1* mutation primarily affected leaflet expansion, rather than leaflet initiation, and the results from *Fragaria vesca* appear consistent with that finding. On the other hand, our results emphasise the importance of *Crd*/*PsYUC1* for the initiation of lateral organs in pea compound leaves. Previously, Zhao et al. [1] postulated a conserved role for *Crd/PsYUC1* and *LLS1* in vein development, but a difference between the two genes in the regulation of leaflet growth.

In pea and *Fragaria vesca*, *YUCCA* mutations reduce the number of leaflet serrations. An inspection of the figures in [1] indicates that this is also the case for the *lls1-1* mutation in *Medicago*. This reduction in serration might be related in some way to the reduced venation of *YUCCA* mutants, since a connection has been made between venation and the “teeth” of leaves [35]. Interestingly, both serrations and leaflets originate from the maxima of auxin action [36].

In general terms, our mutant-based studies have shown that *Crd*/*PsYUC1* is required for the normal degree of complexity of the shoot, the leaves comprising the shoot, and the leaflets comprising the leaves. This gene promotes the formation of leaves on the main stem, of lateral organs on those leaves, and of serrations on the flanks of the leaflets.

*Pisum* tendrils coil in response to mechanical stimulation of their ridged, abaxial surface [37,38,39] and also in response to treatment with auxin [40,41]. Quantification of the IAA concentration in tendrils after stimulation showed a significant increase during the coiling phase [40]. The reduced coiling of *crd* mutant tendrils noted here is an indication that *Crd*/*PsYUC1* plays a role in this sensory phenomenon. The *crd* mutants and their corresponding WTs are a genetic resource that can be exploited for future detailed physiological experiments on tendril coiling.

Even though pea flowers differ markedly from those of Arabidopsis and petunia, *yucca* mutations result in floral deformities in these three species, as well as in *Fragaria vesca* [5]. In pea, floral organ identity was disrupted in the *crd* mutants; for example, the stamen whorl sometimes contained petal-like structures. Dorsal, lateral, and ventral floral petal identities can be distinguished in the zygomorphic pea flower and several genes are known to regulate pea floral organ identity [12,42]. Whether *Crd*/*PsYUC1* affects the production (e.g., transcription, translation, or stability) of floral organ identity regulators or the correct placement of floral organ primordia, which are subsequently acted on by identity regulators, remains to be investigated.

The *crd* mutations reduced the number and length of lateral roots compared to the WT, especially the initiation of lateral roots in the zone immediately below the germinating seed (Figure 5). These *crd* root phenotypes are reminiscent of the auxin-deficient single-*yuc* mutant (*AtYUC8*) root systems recently reported by Jia et al. [43]; however, those effects were manifest only in nitrogen-deficient systems, whereas in our study, the nutrients were not limiting.

In *Arabidopsis* and other model species, the leaves and flowers are thought to originate from the auxin maxima on the flanks of the shoot apical meristem [17,44]. DeMason and Polowick [45] showed that in pea, the primordia that give rise to leaflets and tendrils on the flanks of the incipient leaf axis are also sites of maximum auxin action. In this respect, the developing compound leaf recapitulates the main stem.

There has been much debate over the relative importance of auxin transport and auxin biosynthesis in establishing auxin maxima and minima [17,46,47]. We suggest that in pea leaves, flowers, and roots, reducing auxin biosynthesis disrupts the pattern of auxin maxima and minima, and thereby, organ initiation. This contrasts with the theory, developed for the roots, that auxin directional transport can largely compensate for any reduction in auxin biosynthesis by establishing auxin maxima even when the biosynthetic supply of auxin is reduced [48]. In *crd* mutants, the auxin transport system cannot fully restore organ initiation, even though the overall reduction in auxin content is relatively modest in the shoot [19] and root (Figure S6). It is possible, also, that reduced auxin biosynthesis in the *crd* mutants leads to disrupted auxin transport, since there is thought to be a positive feedback relationship between auxin content and auxin transport [49].

Won et al. [50] suggested that the modest reduction in IAA content in *yuc* mutants might be due to an artificial inflation of that level in mutant extracts via the breakdown of the IAA precursor, IPyA, during the purification procedure. This was investigated by Gélinas-Marion et al. [51], who found that while IPyA does indeed break down in extracts, inflating the IAA pool size, this does not explain the weak effect of *crd* mutations on IAA content. Rather, it appears that since the content of IAA-Asp is reduced to a greater extent than IAA itself in *crd* mutant shoots [19] and roots (Figure S6), a homeostatic mechanism contributes to the larger-than-expected IAA pool in the mutants. Interestingly, in a *Brachypodium distachyon* mutant impaired in the tryptophan to IPyA step, an up-regulation of YUCCA gene expression is suggested to actually increase the IAA content of the root elongation zone, and consequently, root elongation [9]. (The IAA content of root tips was not affected.) Here, we suggest that in pea, a down-regulation of the step IAA to IAA-Asp operates to maintain the IAA content in mutant root tips.

The importance of IAA-Asp has been highlighted recently [52,53], with the finding that this compound is next converted into an inactive form, oxIAA-Asp, by DAO enzymes. Until very recently, whether or not this inactivation step occurs in pea was unknown. Here, we identify oxIAA-Asp and report that the pea genome contains a single *DAO*-like gene. The apparent absence of other *DAO1* genes, as well as the strong phenotype resulting from the virus-induced gene silencing of PsDAO1 [54], strongly implies that it is this protein that is responsible for the formation of oxIAA-Asp in pea.

## 5. Conclusions

In pea, the *Crd*/*PsYUC1* gene promotes the initiation of leaves, leaflets, tendrils, flower components, and lateral roots, and we propose that it is responsible for the supply of IAA at the primordial stages of organ formation. Within pea leaves, this role of *Crd*/*PsYUC1* recapitulates the role of *YUC1* and *YUC4* in the initiation of leaves on the main stem of Arabidopsis [17]. Secondly, organ identity is influenced by *Crd*/*PsYUC1*, which promotes tendril formation and affects stamen/petal identity. Thirdly, *Crd*/*PsYUC1* plays a role in cellular differentiation within organs, particularly in the venation and serration of leaflets.

## Figures and Tables

**Table 1:** Characteristics of compound leaves produced by WT-3 and *crd-3* plants up to and including node 13. n > 11. Tiny leaflets are included in “Number of leaflets per plant”. These numbered only 1.5 ± 0.4 per plant; all in mutant plants. Data are means ± SE; for each characteristic, the two means differ at the *p* < 0.001 level.

Genotype	Number of Leaflets Per Plant	Number of Tendrils Per Plant	Number of Protuberances and Hair-like Organs Per Plant	Total Number of Leaflets, Tendrils, Protuberances, and Hair-like Organs Per Plant
WT-3	28.4 ± 0.5	35.5 ± 0.8	0	63.9 ± 1.2
*crd-3*	19.3 ± 0.7	21.0 ± 1.0	5.0 ± 0.8	45.2 ± 1.2

## Data Availability

Data are contained within the article and Supplementary Materials.

## Supplementary Materials

The following supporting information can be downloaded at: https://www.mdpi.com/article/10.3390/genes14122115/s1, Figure S1. Structures of the WT pea compound leaf and flower; Figure S2. Leaflet area plotted against node number for WT-1 and *crd-1* plants. Number of nodes formed in mutant and WT plants; Figure S3. Leaves of *crd* mutants and corresponding WT plants; Figure S4. Flower structure of the *crd-4* mutant, compared to the corresponding WT-4; Figure S5. Root growth traits of WT-3 and *crd-3* plants on the Caméor background; Figure S6. IAA and IAA-Asp levels in lateral root tips of WT-3 and *crd-3* plants (Caméor background); Figure S7. Chromatograms for diagnostic mass transitions for oxIAA-Asp; Figure S8. NMR of oxIAA-Asp synthesised as in Materials and Methods section; Figure S9. Alignment of amino acid sequences from DAO proteins from a range of angiosperm plant species; Figure S10. Phylogenetic tree of angiosperm DAO proteins; Table S1. Characteristics of compound leaves produced by WT-1 and *crd*-1 plants; Table S2. *PsYUC* gene nomenclature.
