# Supplementary material for: Plant Development in the Garden Pea as Revealed by Mutations in the Crd/PsYUC1 Gene
Source: Genes (Basel). 2023 Nov 23;14(12):2115. doi: 10.3390/genes14122115 (PMC10742580; doi:10.3390/genes14122115)
Supplement: Supplementary file 1 [file genes-14-02115-s001.zip › genes-2442333-supplementary.pdf]

Figure S1. Structures of the WT pea compound leaf and flower. A. The leaf shown has four leaflets (a proximal pair and a distal pair) and five tendrils. B. The flower shown has five sepals, ten stamens (one free), one carpel and five petals; one banner, two alae and two carina petals. Scale bars = 10 mm. (Line: JI2822).

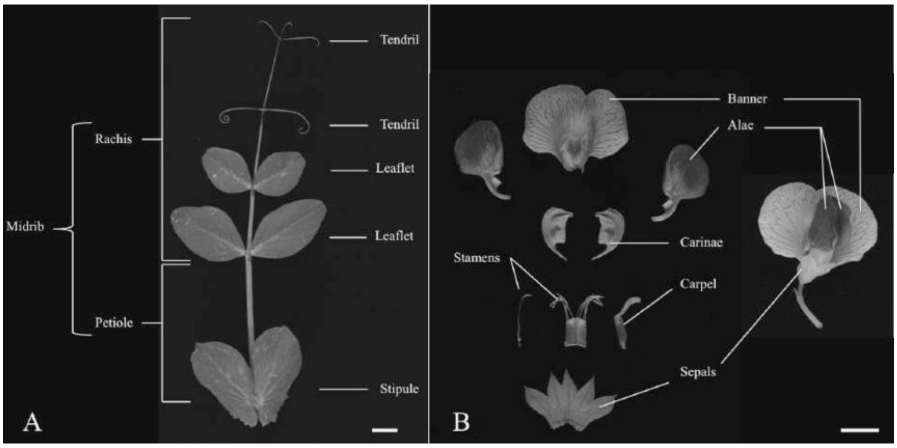

Figure S2. a), Leaflet area plotted against node number for WT-1 and *crd-1* plants. Data are means ± S.E. b), Number of nodes formed by *crd* mutant and accompanying WT plants 38 days after sowing. Data are are means ± S.E; n>8. Asterisks indicate a significant difference (P<0.05) between the mutant and relevant WT.

a

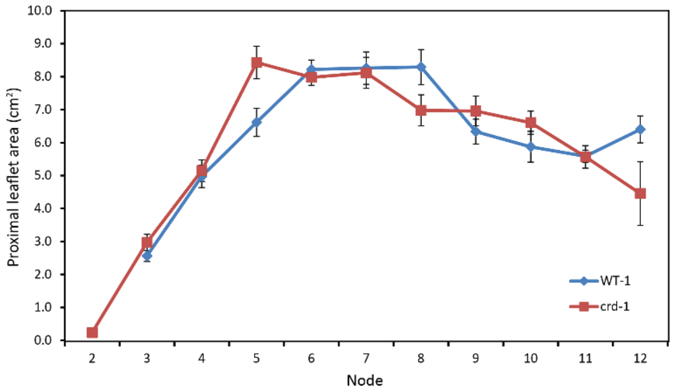

b

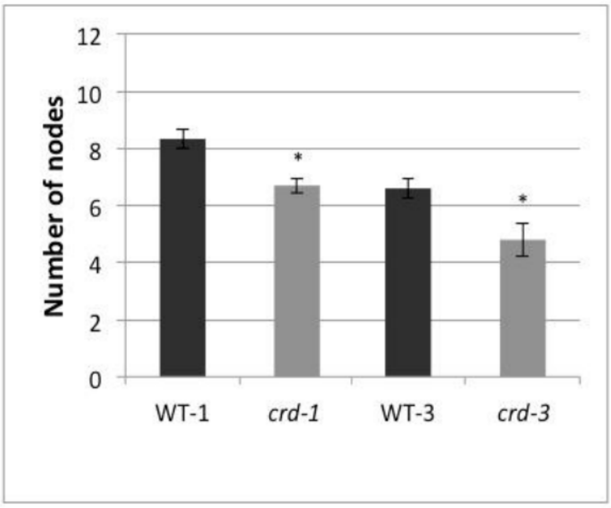

Figure S3. Leaves of WT-3 (top) and the *crd-3* mutant; scale bar = 20 mm. Leaves are from node 7.

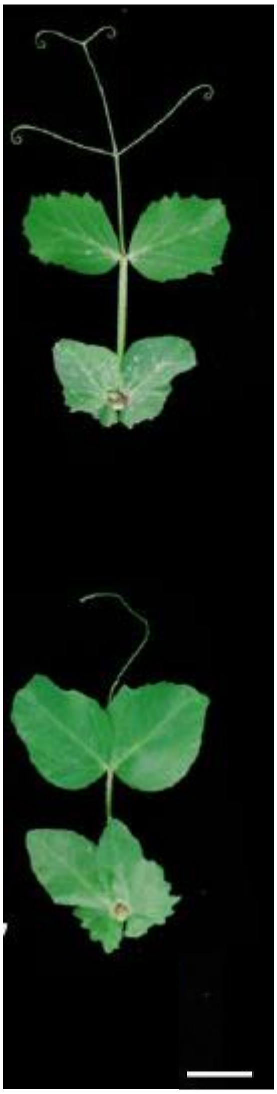

Figure S4. Flowers of WT-4 and the *crd-4* mutant, in a tall background. For each genotype, shown are the 1st and 2nd flowers from a single inflorescence. Scale bar = 10 mm.

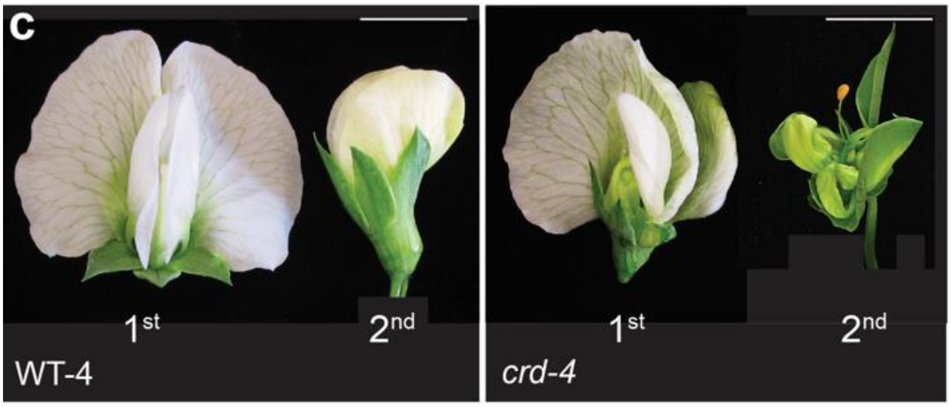

Figure S5. Root growth traits of WT-3 and *crd-3* plants in the Caméor background. a) Taproot length. b) Mean of the average length of the 6 longest lateral roots of each plant (n = 10; P<0.01) and c) Number of lateral roots (n = 10; p<0.01). Results are means ± S.E. and asterisks denote significant differences.

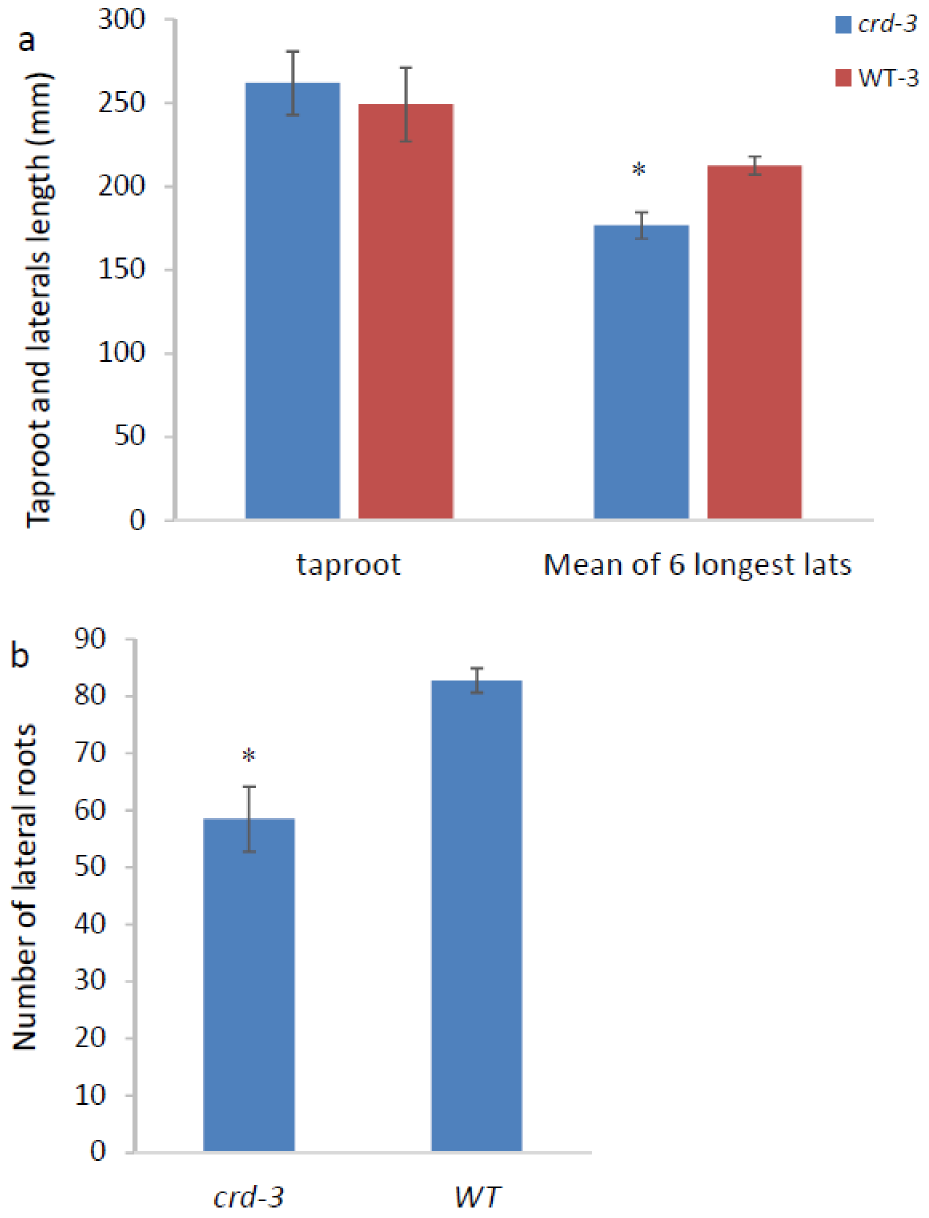

Figure S6. Content of IAA (a) and IAA-Asp (b) in lateral root tips (2 mm) of *crd-3* mutant and WT-3 plants on the Caméor background (n = 5). Results are means ± S.E. and the asterisk denotes a significant difference (P<0.05).

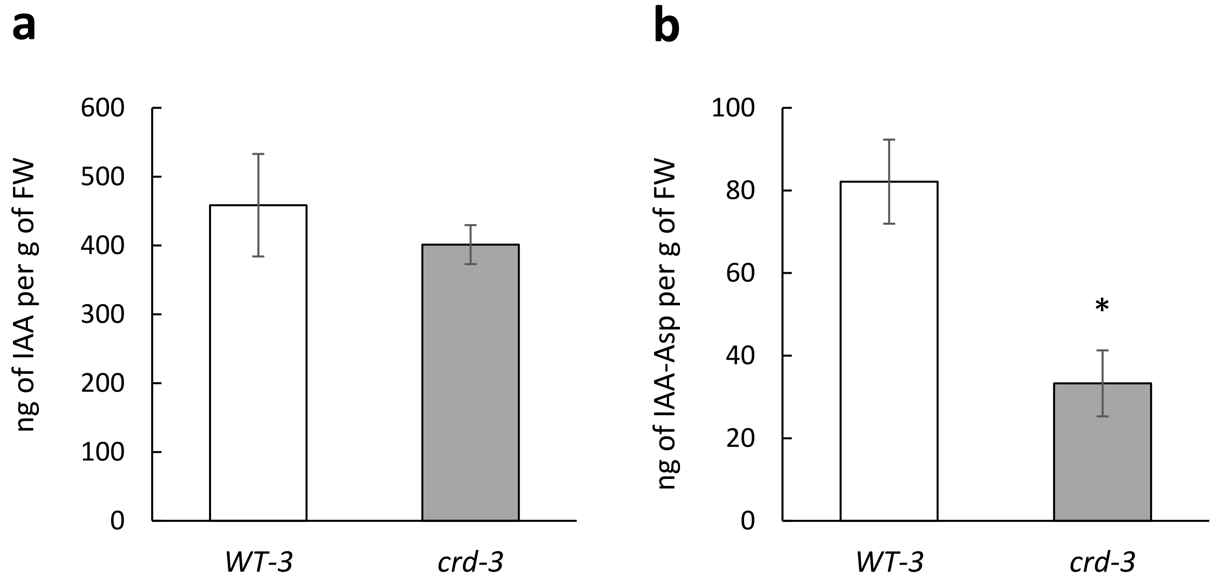

Figure S7. Chromatograms for diagnostic mass transitions for oxIAA-Asp. a) Authentic standard synthesised as in Materials and Methods. b) Extract from young peas seeds. The LC retention time and multiple reaction monitoring transition profiles for oxIAA-Asp from the seed extract exactly matched those of synthesised oxIAA-Asp, with the two diastereomers of this compound partially separable by LC. The proportion of the two diastereomers is known to change during purification [52].

a

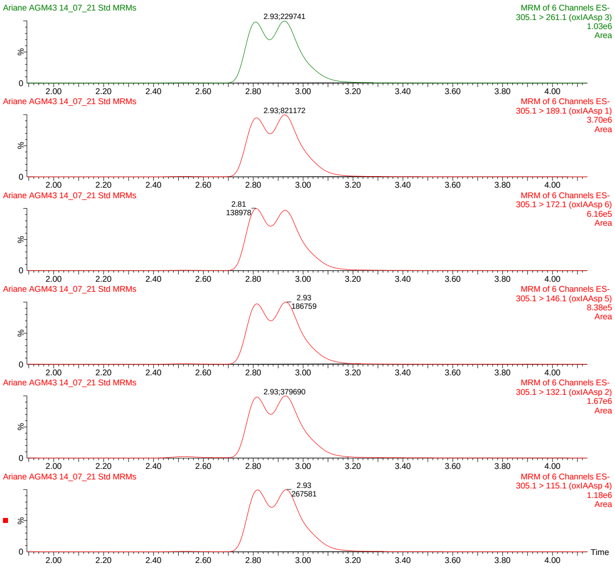

b

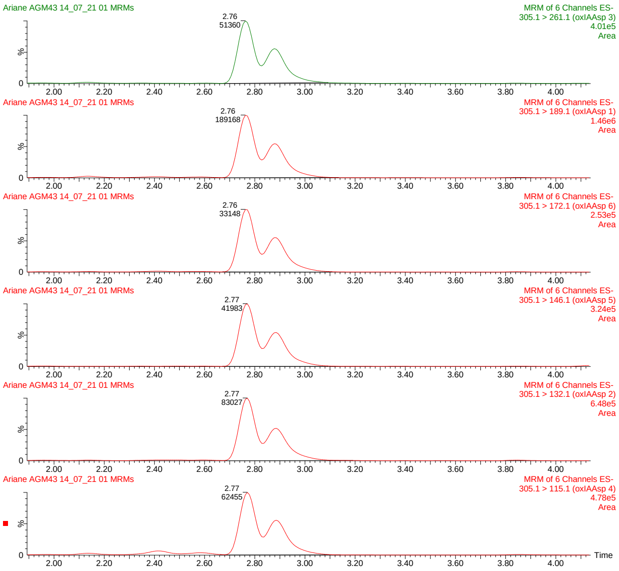

Figure S8. Confirmation of identity of the oxIAA-Asp synthesised as in Materials and Methods. (a) ^1H and (b) ^{13}C NMR data for oxIAA-Asp run in CD_3OD.

a

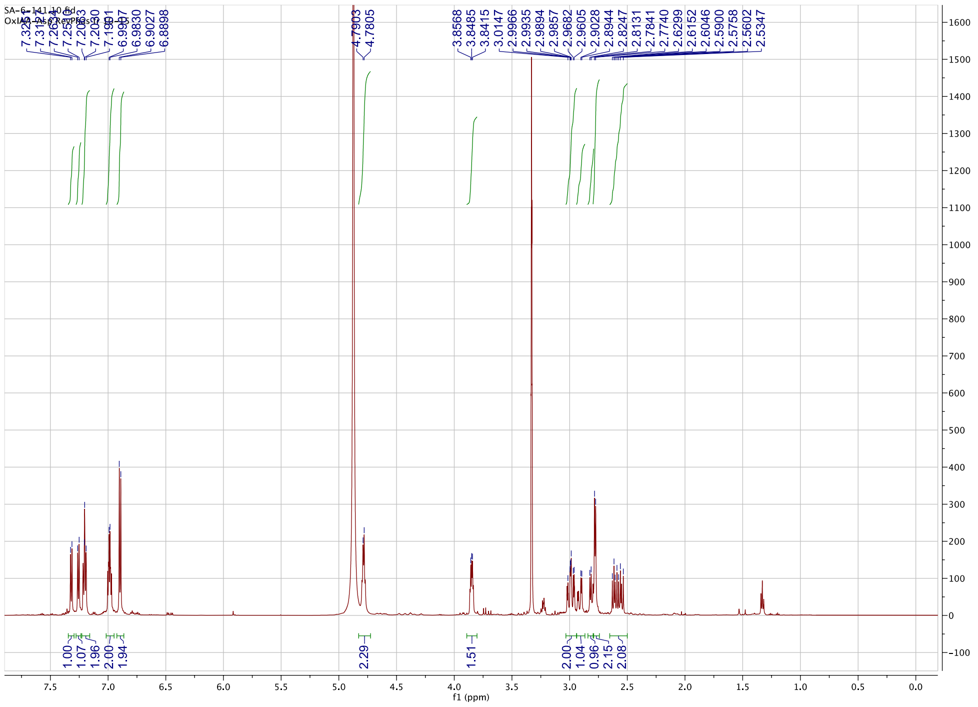

b

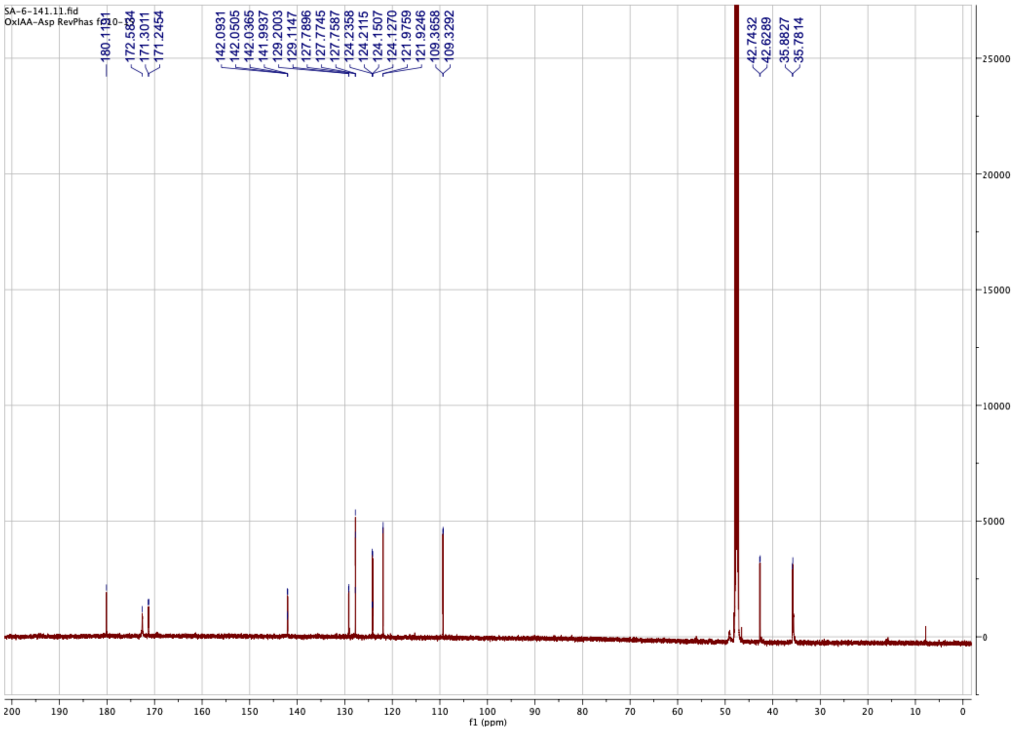

Figure S9. Evidence that *Psat2g146920* (PsDAO1) encodes a DAO enzyme. Alignment of amino acid sequences from DAO proteins from a range of angiosperm plant species. Protein identifiers: OsDAO: Os04g0475600; ZmDAO2: GRMZM2G127232; ZmDAO1: GRMZM2G121700; AtDAO1: AT1G14130; AtDAO2: AT1G14120; NtDAO1: A0A1S3XSG2; PvDAO1: Phvul.002G120100; PvDAO2: Phvul.002G120000; MtDAO2: Medtr5g025050; PsDAO1: Psat2g146920; MtDAO1: Medtr5g025090; LjDAO3: LotjaGi2g1v0351100.1; LjDAO1: LotjaGi2g1v0351600.1; LjDAO2: LotjaGi2g1v0351400.1.

Psat2g146920 contains both the dioxygenase and 2OG Fe (II) domains as well as the DAO motif proposed in [29]. The three shades of blue, from light to dark, indicate an increasing degree of conservation.

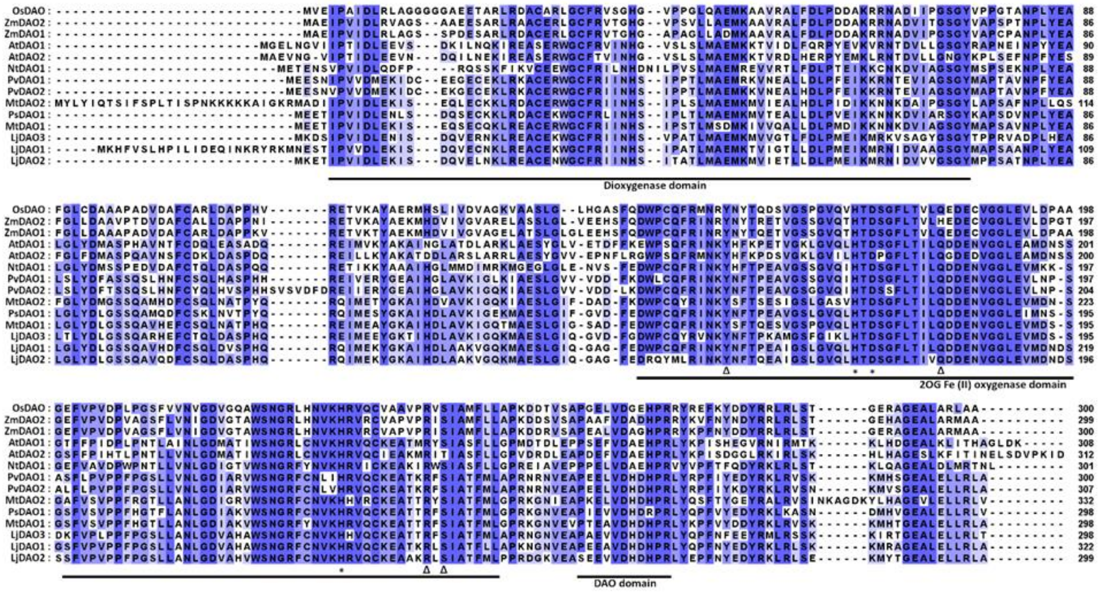

Figure S10. Phylogenetic tree of angiosperm DAO proteins, including PsDAO1. Tree topology was inferred by using the Maximum Likelihood method and JTT matrix-based model in MEGA [30]. This was applied to a ClustalO alignment of the 2OG Fe (II) oxygenase domains (positions corresponding to 173-244 of OsDAO in Fig S9) from 17 DAO protein sequences including those listed in Fig S9 and AtDAO1 orthologues from *Marchantia polymorpha* subsp. Ruderalis (MpDAO1; OAE33245.1) and *Solanum lycopersicum* (SlDAO1; XP_004233276.1 and SlDAO2; XP_004233278.1). The tree with the highest log likelihood (-2059.50) is shown. The percentage of trees in which the associated taxa clustered together is shown next to the branches (1000 Bootstrap replicates). The tree is drawn to scale, with branch lengths measured in the number of substitutions per site.

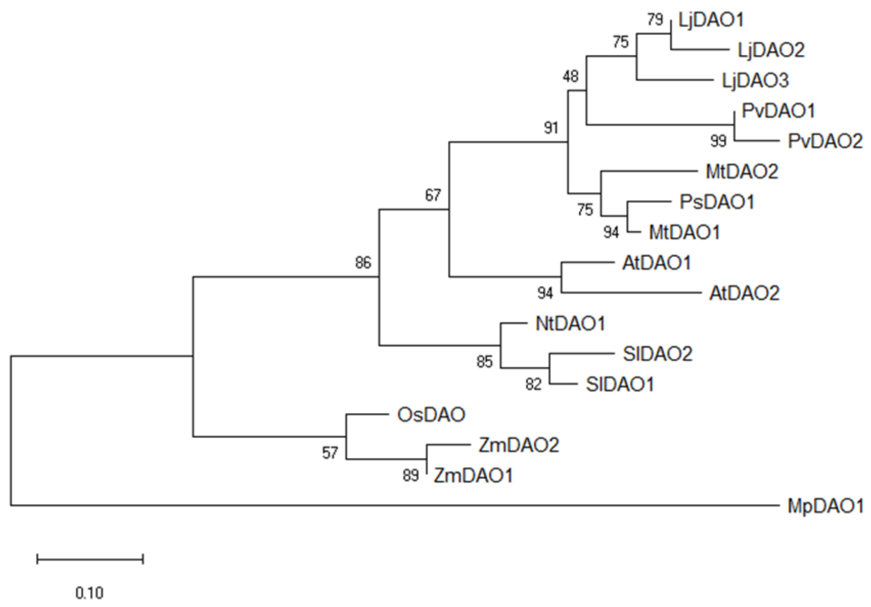

Table S1. Characteristics of compound leaves produced by WT-1 and *crd-1* plants. Leaves from nodes 13 to 19 inclusive were analysed. N = 13 for both groups; data are means ± S.E. Tiny leaflets are not included in "Number of leaflets per plant"; these numbered only two, both in mutant plants.

Genotype	Number of leaflets per plant	Number of tendrils per plant	Number of protuberances per plant	Total number of Leaflets, tendrils and protuberances per plant
WT1	28.0 ± 0.5	37.2 ± 0.8	0	65.2 ± 1.1
crd-1	21.8 ± 0.7	20.2 ± 0.8	1.9 ± 0.4	43.8 ± 1.0

Table S2. *PsYUC* gene nomenclature

Gene (Kreplak *et al.*, 2019) [26]	Tivendale *et al.* (2010) [21] nomenclature	Davis *et al.* (2020) [31] nomenclature	Kaur *et al.* (2021) [32] nomenclature	Chabikwa *et al.* (2019) [33] nomenclature
Psat6g030600	*PsYUC1* (*Crd*)	*PsYUC1*		*PsYUC4*
Psat5g023680	*PsYUC2*	*PsYUC2*	*PsYUC6-2*	
Psat1g042120		*PsYUC3*	*PsYUC2*	
Psat5g082680		*PsYUC4*		
Psat3g053880		*PsYUC5*		
Psat3g024720		*PsYUC6*	*PsYUC10*	
Psat5g060240		*PsYUC7*		
Psat3g024760		*PsYUC8*		
Psat6g034040			*PsYUC6-1*	
